# Interaction between Tat and Drugs of Abuse during HIV-1 Infection and Central Nervous System Disease

**DOI:** 10.3389/fmicb.2015.01512

**Published:** 2016-01-11

**Authors:** Monique E. Maubert, Vanessa Pirrone, Nina T. Rivera, Brian Wigdahl, Michael R. Nonnemacher

**Affiliations:** ^1^Department of Microbiology and Immunology, Drexel University College of MedicinePhiladelphia, PA, USA; ^2^Center for Molecular Virology and Translational Neuroscience, Institute for Molecular Medicine and Infectious Disease, Drexel University College of MedicinePhiladelphia, PA, USA

**Keywords:** HIV-1, Tat, drugs of abuse, ART, HAND, blood-brain barrier, CNS cells

## Abstract

In many individuals, drug abuse is intimately linked with HIV-1 infection. In addition to being associated with one-third of all HIV-1 infections in the United States, drug abuse also plays a role in disease progression and severity in HIV-1-infected patients, including adverse effects on the central nervous system (CNS). Specific systems within the brain are known to be damaged in HIV-1-infected individuals and this damage is similar to that observed in drug abuse. Even in the era of anti-retroviral therapy (ART), CNS pathogenesis occurs with HIV-1 infection, with a broad range of cognitive impairment observed, collectively referred to as HIV-1-associated neurocognitive disorders (HAND). A number of HIV-1 proteins (Tat, gp120, Nef, Vpr) have been implicated in the etiology of pathogenesis and disease as a result of the biologic activity of the extracellular form of each of the proteins in a number of tissues, including the CNS, even in ART-suppressed patients. In this review, we have made Tat the center of attention for a number of reasons. First, it has been shown to be synthesized and secreted by HIV-1-infected cells in the CNS, despite the most effective suppression therapies available to date. Second, Tat has been shown to alter the functions of several host factors, disrupting the molecular and biochemical balance of numerous pathways contributing to cellular toxicity, dysfunction, and death. In addition, the advantages and disadvantages of ART suppression with regard to controlling the genesis and progression of neurocognitive impairment are currently under debate in the field and are yet to be fully determined. In this review, we discuss the individual and concerted contributions of HIV-1 Tat, drug abuse, and ART with respect to damage in the CNS, and how these factors contribute to the development of HAND in HIV-1-infected patients.

## Introduction

**Figure 1:**
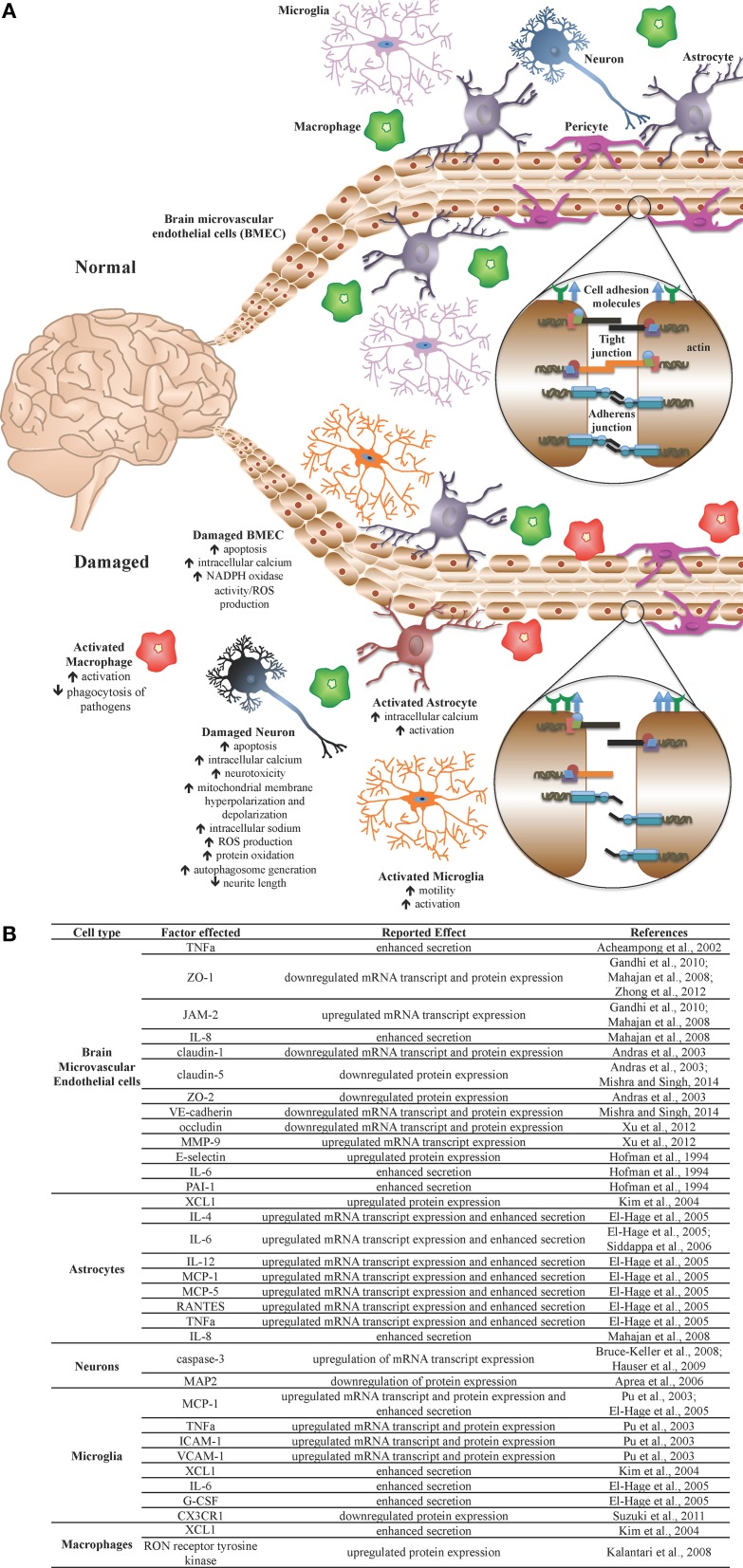
**The blood-brain barrier (BBB) under normal and pathologic conditions**. The BBB is a network of microvasculature composed primarily of endothelial cells, astrocytes, and pericytes and functions as a selective, semi-permeable barrier, thus maintaining central nervous system (CNS) homeostasis and regulating communication between the CNS and the periphery. The semi-permeable nature of the BBB, including cellular transmigration across the barrier, is regulated through the expression of tight junction complexes, adherens junction complexes, and cell adhesion molecules. **(A)** Under normal homeostatic conditions, CNS component cells of the BBB, as well as CNS immune regulators, are healthy and BBB integrity is properly maintained. During infection or other assault, activation of select CNS cells results in a pro-inflammatory environment, yielding cellular, and tissue injury, including altered expression of cell adhesion molecules, adherens junction proteins, and tight junction proteins, resulting in BBB compromise. **(B)** During HIV-1 infection, Tat protein mediates detrimental effects on specific cells, including CNS component cells and CNS immune regulators, altering the activation status, and molecular profiles of several cell types, resulting in a pro-inflammatory environment, and associated damage to the BBB. It is theorized that Tat is thus an important viral factor in the incidence and progression of HAND in HIV-1-infected patients.

The face of the HIV-1 pandemic has evolved from a progressively advancing life-threatening disease in the absence of effective therapies to a manageable chronic clinical condition with the development of effective combination antiretroviral therapy (ART). As a result of more effective therapeutic control of HIV-1 disease for prolonged periods of time across the infected population, many comorbid conditions have decreased in prevalence while some have increased. To this point, there has been a dramatic reduction in the prevalence of the more severe forms of HIV-1-associated neurocognitive disorders (HAND) including the most severe form, the progressively debilitating HIV-1-associated dementia (HAD), that was commonly observed at end-stage HIV-1 infection in the absence of effective ART. However, there has been an increase in the milder forms of disease encompassing a spectrum of neurological symptoms, ranging from a clinically asymptomatic neurocognitive impairment (ANI) to more symptomatic presentations of cognitive impairment (Wadia et al., 2001; Antinori et al., 2007). The mechanisms by which HIV-1 infection promotes neuropathogenesis is based on viral entry into the central nervous system (CNS) early during the course of infection by breaching of the blood-brain barrier (BBB) followed by a series of events that center around the neurotoxic activity of a number of HIV-1 proteins including gp120, Tat, Vpr, and likely others, along with alterations in CNS homeostasis that involve the metabolic integrity of the blood-brain barrier and metabolism of astrocytes, perivascular macrophages, and resident microglial cells. These are all events that lead to disruption of neuronal physiology and increasing levels of death in this critical cell population (Toborek et al., 2005; Rao et al., 2014) (Figure 1).

HIV-1 proteins (gp120, Tat, Vpr, and Nef) are released from infected cells, either through secretion or after lysis of the cell, throughout the course of infection and perpetuate an ongoing burden in a number of tissues, including the CNS, resulting in toxicity and damage, regardless of ART (Wang et al., 2006; Strazza et al., 2011; Gresele et al., 2014). Of these, Tat (the transactivator of transcription) is a viral protein, 101 amino acids in length, known to be made and secreted early and continuously by HIV-1-infected cells in the CNS throughout infection and has been implicated in mediating and altering the functions of several host factors, disrupting the molecular and biochemical balance of numerous pathways, thus contributing to cellular toxicity, dysfunction, and death (Ensoli et al., 1993; Chang et al., 1997; Rappaport et al., 1999; Li et al., 2009). Tat functions as the primary viral transcription factor, binding and altering the function of a number of cellular players in the host transcriptional machinery (Frankel and Young, 1998; Friedrich et al., 2011; Ramakrishnan et al., 2012). The transactivator has been shown to consist of six domains that have been linked experimentally to a number of Tat-mediated consequences, including both intracellular- and extracellular-triggered events (Jeang et al., 1999; Debaisieux et al., 2012; Li et al., 2012).

Extracellular Tat has been shown to adversely impact a number of cell types, exhibiting particularly negative effects on cells of the CNS, including neurons, astrocytes, brain microvascular endothelial cells (BMEC), and microglia, as well as macrophages. Extracellular Tat protein is quickly and efficiently taken up by uninfected neurons (Kolson et al., 1994) and astrocytes (Ma and Nath, 1997) exerting direct and indirect consequences on these and neighboring CNS cells. These harmful outcomes include neuronal toxicity and dysfunction via mitochondrial membrane hyperpolarization and aberrant synaptic signaling (Chauhan et al., 2003; Norman et al., 2007), altered dendritic arborization and morphology (Bruce-Keller et al., 2003; Aprea et al., 2006), and deregulation of epigenetic modulators (Saiyed et al., 2011). In addition, Tat exposure has also been shown to mediate apoptosis (Acheampong et al., 2002; Kim et al., 2003), alter molecular permeability (András et al., 2003; Mahajan et al., 2008; Gandhi et al., 2010; Mishra and Singh, 2014), and enhance secretion of pro-inflammatory cytokines (Acheampong et al., 2002; Woollard et al., 2014) from BMECs, a primary component of the BBB.

These issues are further complicated by naturally occurring comorbid diseases and conditions, the prevalence of which appear to be accelerated or augmented in HIV-1-infected patients, including increased permeability of the BBB and an enhanced chronic pro-inflammatory state in the host, as compared to the general population (Weiss et al., 2009; Scott et al., 2011; Pirrone et al., 2013; Nasi et al., 2014). Moreover, host genetics have been shown to play an irrefutable role in the grand scheme of disease severity (if not incidence) and rate of progression of neurocognitive deficits in all patients, regardless of HIV-1 status, which is additionally confounded by lifestyle, including but not limited to diet and exercise habits, medicinal intake, therapy, and indulgence in recreational drugs (Nath et al., 2002; Liu et al., 2012; Nasi et al., 2014; Smith et al., 2014).

Across four decades of increasingly effective therapies, the HIV-1 protein Tat has been shown to play an important role in viral replication and pathogenesis as both an intracellular and extracellular protein. At present, in ART-naïve patients experiencing varying degrees of immunological control, as well as patients well-suppressed by ART, and patients experiencing less than ideal responses to ART for numerous reasons, Tat has continued to be a focus as a protein that may be produced and released from cells in the absence or presence of infectious virus production (Falkensammer et al., 2007; Mediouni et al., 2012) with subsequent impact on immunologic and neurologic function, to the detriment of the host. It has been suggested that this may be one of the underlying mechanisms of HAND (involving Tat production and release, leading to alterations in inflammatory molecule production by interacting with cellular gene promoters and cellular receptors to induce apoptosis and cell death) (Rappaport et al., 1999; Bagashev and Sawaya, 2013). With respect to drugs of abuse, it is widely known that drug use is widespread in the HIV-1-infected community. It has also been shown in a number of HIV-1-infected patient cohorts that there are preferences with respect to the type of drugs used that have been associated with the region of the United States or part of the world wherein a patient resides. Consequently, these preferences complicate our ability to understand how select drugs interact with HIV-1 and its gene products. Furthermore, regional differences in substance abuse profiles, and methodologies used to define the types of substances used (e.g., self-reporting, medical history, blood and/or urine analysis, as well as hair follicle analysis) further complicates studies focused on understanding the use of any single substance (Levine et al., 2014; Holtz et al., 2015; Rosinska et al., 2015). These considerations are further impacted by the fact that a majority of HIV-1-infected individuals can test positive at any given clinical visit for three or more substances that often include tobacco, alcohol, cocaine, heroin, cannabis, and many others (Nishijima et al., 2013; Chang et al., 2014; Huang et al., 2014; Ti et al., 2014). Given these considerations, understanding regional drug use demographics, frequency of use, and blood levels are important factors to consider in the data analysis phase of experimentation and in formulating conclusions.

## Tat and drugs of abuse in the CNS

Clinical reports, as well as experiments involving both *in vitro* and *in vivo* investigations, have been instrumental in defining the factors and pathways implicated in neurocognitive compromise and their involvement in HAND. Drug use is a well-known confounding factor that contributes to neurocognitive impairment and the development of dementia. Within HIV-1-infected populations, the widespread use of recreational drugs (including opiates, amphetamines, cocaine, and ethanol) has been shown to adversely impact the incidence and severity of HAND (as well as other HIV-1-associated diseases), as compared to non-users (Nath et al., 2002; Green et al., 2004; Sharma and Ali, 2006; Theodore et al., 2007; Silverstein et al., 2011; Hauser et al., 2012; Nair and Samikkannu, 2012; Rao et al., 2014). In particular, the HIV-1 Tat protein has been identified, both *in vitro* and *in vivo*, to perform in an additive or synergistic manner in a number of experimental Tat—drug abuse co-exposure models (Nath et al., 2002; Li et al., 2009).

### Tat and opiates

Of the four opioid receptors currently described in the literature (i.e., delta, kappa, mu, and opioid-receptor-like1), the mu opioid receptor is the most common target of currently available opioids, both in the clinic and on the streets, and is expressed in many tissues throughout the body including the CNS (Al-Hasani and Bruchas, 2011; Feng et al., 2012). Morphine, which is administered to patients as part of many pain management regimens, is also a metabolic derivative of heroin, and binds the mu opioid receptor with high affinity (Bell, 2014). The singular and synergistic effects of HIV-1 Tat and opiates (specifically morphine) on select cells of the CNS have been demonstrated experimentally both *in vitro* and *in vivo*, providing biochemical and cellular correlates for observed clinical manifestations of HAND.

Utilization of an *in vitro* BBB model comprised of primary human BMEC (hBMEC) and astrocytes in co-culture demonstrated a decrease in barrier tightness coupled to a parallel increase in immune cell transmigration as a result of either Tat or morphine exposure, and BBB permeability was further exacerbated by co-exposure to both agents simultaneously (Mahajan et al., 2008). Exposure to either morphine or Tat also resulted in diminished mRNA expression of the tight junction proteins (TJP) occludin and zona occludens-1 (ZO-1), and a concomitant increase in the cell adhesion molecule (CAM) junctional adhesion molecule-2 (JAM-2) mRNA expression in hBMEC, and these events were amplified by co-exposure to both compounds (Mahajan et al., 2008). Mono-exposure of the hCMEC/D3 BMEC line to Tat also resulted in the enhanced nuclear translocation of ZO-1 within exposed cells (Zhong et al., 2012), and Tat also downregulated mRNA and protein expression of occludin of exposed primary hBMECs (Xu et al., 2012). In addition, the proliferative capacity of primary murine oligodendroglial progenitors was impeded (Hahn et al., 2012), and activation of caspase-3 in primary murine oligodendrocytes was augmented (Hauser et al., 2009), by exposure to either morphine or Tat, and these effects were further potentiated by co-exposure. Caspase-3 activation and apoptosis were also upregulated in primary murine glial precursor cells by mono-exposure to either Tat or morphine, *in vitro* (Buch et al., 2007).

Primary murine astrocytes or microglia (Pu et al., 2003; El-Hage et al., 2005, 2008; Bokhari et al., 2009), as well as human U373 MAGI astrocytes and primary human monocytes (Siddappa et al., 2006) exposed to Tat, and primary hBMEC exposed to Tat or morphine (Mahajan et al., 2008) enhanced secretion and mRNA transcript expression of a number of pro-inflammatory cytokines (including IL-1B, IL-6, IL-8, MCP-1, and TNFα), which was further augmented in both astrocytes and BMEC by co-exposure of cells with both Tat and morphine. Furthermore, exposure of primary murine microglia to either morphine or Tat protein alone prompted nominal increases in expression of CCR5, and differences in morphology and activation status (Turchan-Cholewo et al., 2008; Bokhari et al., 2009), as well as aberrant regulation and expression of opioid receptors (Turchan-Cholewo et al., 2008; Bokhari et al., 2009), which were all exacerbated by co-exposure (Turchan-Cholewo et al., 2008; Bokhari et al., 2009). In addition, direct exposure to either morphine or Tat enhanced migration of primary murine microglia (Suzuki et al., 2011), while treatment with conditioned media from Tat- or morphine-exposed astrocytes also enhanced migration of the N9 murine microglia cell line (El-Hage et al., 2006) *in vitro*. Notably, this escalation in microglial cell migration was further increased by direct co-exposure to morphine and Tat (Suzuki et al., 2011) or to media derived from co-exposed astrocytes (El-Hage et al., 2006).

*In vitro* exposure to Tat increased intracellular sodium- and calcium-ion concentrations in primary murine striatal neurons (Fitting et al., 2014), as well as mitochondrial membrane depolarization in primary human and murine neurons, as well as in the SHSY5Y human neuronal cell line (Malik et al., 2011; Fitting et al., 2014), and these effects were dramatically amplified by co-exposure with morphine, demonstrating increased Tat-mediated neurotoxicity in the presence of opiates. Additionally, an increase in apoptosis, as well as enhanced generation of reactive oxygen species (ROS) by exposure to either Tat or morphine alone, was further enhanced by co-exposure in both primary human neurons and in SHSY5Y cells (Malik et al., 2011; Suzuki et al., 2011). Interestingly, the neurotoxic consequences of co-exposure were attenuated in primary murine neurons in the presence of exogenous fractalkine treatment (Suzuki et al., 2011), a neuroprotective chemokine shown to prevent apoptosis of Tat-exposed primary rat neurons *in vitro* (Tong et al., 2000) and constitutively produced in human brain tissue (Wang et al., 2006), but downregulated by co-exposure to Tat and morphine (Suzuki et al., 2011), suggesting the potential for adjunctive fractalkine-enhancing therapy in HIV-1-infected patients with HAND.

With respect to *in vivo* experimental results, utilization of a doxycycline-inducible Tat-transgenic mouse model under control of the human glial acidic fibrillary protein (GFAP) promotor (allowing for brain-specific expression of Tat) was shown to exhibit a higher percentage of activated microglia and macrophages, as well as astrocytes, and this activation was further potentiated by morphine treatment (through the implantation of a slow-release subcutaneous pellet; Bruce-Keller et al., 2008). Furthermore, neurons from Tat-transgenic mice displayed an approximately 10-fold upregulation of caspase-3 expression, which was additionally boosted by treatment with morphine (Bruce-Keller et al., 2008; Hauser et al., 2009). *In vivo* induction of Tat in these transgenic mice also instigated aberrant morphological changes in oligodendrocytes, including diminished length and number of cellular processes, as well as the presence of dendrite swelling and phenotypically abnormal cell bodies (Hauser et al., 2009); curiously, morphine exposure alleviated Tat-mediated effects on process lengths, however all other aberrations were exacerbated. Both induction of Tat in transgenics and subcutaneous injection of morphine in Tat-negative mice resulted in decreased proliferative capacity of brain cells, in general, and of undifferentiated and oligodendroglial progenitors, in specific, which was additionally reduced in morphine-treated Tat-transgenic mice (Hahn et al., 2012). Behaviorally, subcutaneous injection of morphine in non-transgenic mice and induction of Tat in transgenic mice individually slowed reaction times and compromised performance of exposed animals in nociception and balance testing (Fitting et al., 2012), demonstrating independent effects of these compounds that can combine to adversely impact general neuro-functional outcomes in HAND.

### Tat and cocaine

Cocaine highjacks dopamine signaling pathways *in vivo* by binding up the dopamine transporter in the synapse, thereby causing increased localized concentration of dopamine and heightened stimulation of receiving post-synaptic neurons, thus exerting its effects which result in the well-known “high” experienced by users (Nestler, 2005; Blaylock and Nader, 2012). The consequences of *in vitro* exposure of CNS component cells to mono- or co-exposure to Tat and cocaine has yielded interesting observations with implications for CNS disease manifested in cocaine-using patients with HAND.

Studies have indicated that exposure of primary rat neurons to Tat alone resulted in a decrease in mitochondrial membrane potential and cellular viability, as well as a concomitant increase in the generation of intracellular ROS and enhanced protein oxidation, all of which were significantly potentiated by co-exposure to cocaine, *in vitro* (Aksenov et al., 2006). In addition, mono-exposure of an *in vitro* BBB primary human BMEC-astrocyte co-culture model to Tat or cocaine resulted in increased BBB compromise and permeability, including an increase in monocyte transmigration across the *in vitro* barrier, as well as alterations in TJP (ZO-1) and CAM (JAM-2) expression (Gandhi et al., 2010); observations which were further exacerbated by exposure to both compounds in tandem.

*In vivo*, both acute (involving a 1-day interval) and chronic (involving 7–14-day intervals) exposure to either cocaine by intra-peritoneal (IP) injection, or intra-accumbal (IA) injection of Tat, differentially deregulated the generation of extracellular dopamine metabolites in the brains of exposed rats, and Tat-mediated effects on metabolite levels were further exacerbated by co-exposure to cocaine (Ferris et al., 2010). IP injection of rats with cocaine alone enhanced neuronal excitability, however, bathing of *ex vivo* pre-frontal cortex pyramidal neurons from animals exposed *in vivo* to chronic (14 days of exposure) cocaine with exogenous Tat further augmented neuronal excitability (Napier et al., 2014), providing credence to an interactive role of cocaine and Tat in observed neurocognitive impairment in cocaine-using HIV-1-infected patients.

Behaviorally, cocaine mono-exposed animals (but not Tat co-exposed animals) displayed a higher level of activity than Tat-only or vehicle-controls, demonstrating diverging behavioral consequences of cocaine use in the context of HIV-1 infection (Ferris et al., 2010). Importantly, pre-exposure of rats to IA-injected Tat amplified the effects of an acute 1-day exposure to intravenous (IV) cocaine, but mitigated the effects of a chronic 14-day exposure of cocaine to sensitization, highlighting differences between acute vs. chronic cocaine use during the course of *in vivo* Tat exposure (Harrod et al., 2008). Furthermore, utilization of a brain-specific doxycycline-inducible Tat-transgenic mouse model under control of the murine GFAP promotor yielded several-fold increases in cocaine-conditioned place preferences by Tat-induced mice after subcutaneous injection of cocaine [cocaine-conditioned place preference being a correlate for the rewarding effects of addictive behavior, defined by the amount of time an animal spends in the cocaine-associated chamber as compared to the pre-cocaine baseline] (Paris et al., 2014). This observation provides evidence that CNS expression of Tat is capable of intensifying cocaine-additive behaviors *in vivo*.

### Tat and amphetamines

Amphetamines function similarly to cocaine in their ability to interfere with dopamine signaling pathways, thus resulting in a build-up of dopamine in the synapse and over-stimulation of post-synaptic neurons resulting in the characteristic “high” experienced by users (Calipari and Ferris, 2013). Both *in vitro* and *in vivo* experiments have been conducted in order to elucidate the consequences of exposure to Tat and/or amphetamines on relevant cells of the CNS, and how these may be involved in the HAND outcomes observed in HIV-1-infected patients who use amphetamines.

*In vitro* studies demonstrate susceptibility of primary rat neurons to apoptosis by exposure to Tat (Theodore et al., 2006a), and of SHSY5Y cells by treatment with methamphetamine (Qi et al., 2011), with cellular viability further compromised by co-exposure to both agents (Maragos et al., 2002; Theodore et al., 2006a; Qi et al., 2011). Co-exposure of primary human neurons to Tat and methamphetamine also increased cell death, which was accompanied by a decrease in mitochondrial membrane potential (Maragos et al., 2002) and characterized by a concomitant increase in the presence of autophagosomes (Qi et al., 2011). In addition, exposure of primary human neuron-astrocyte co-cultures to methamphetamine alone also enhanced secretion of matrix metalloproteinase-1 (MMP-1), whereas exposure to Tat upregulated secretion of both MMP-1 and MMP-2 (Conant et al., 2004), proteases capable of degrading select extracellular matrix components, thus compromising BBB integrity. Transfection of the U87MG human astrocyte cell line with Tat plasmid or exposure of cells to methamphetamine inhibited beta-catenin signaling, which was further diminished by co-exposure to both compounds (Sharma et al., 2011). Furthermore, co-transfection of CHME-5 human microglia cells with LTR- and Tat-containing plasmids exposed to methamphetamine augmented HIV-1 LTR transactivation by Tat, as well as NFκB nuclear translocation, in a dose-dependent manner (Wires et al., 2012), underscoring a potential cooperative relationship of amphetamines and Tat in enhancement of viral replication in HIV-1-infected patients who use amphetamines.

*In vivo* experiments have demonstrated upregulated expression of a number of pro-inflammatory cytokines in response to Tat and amphetamine exposure. Notably, both TNFα secretion (Flora et al., 2003; Theodore et al., 2006a,b; Liu et al., 2014) and mRNA transcript expression (Flora et al., 2003; Theodore et al., 2006a,b; Liu et al., 2014) were upregulated in models utilizing different routes of Tat administration [including intra-striatal (IS) injection (Flora et al., 2003; Theodore et al., 2006a,b; Liu et al., 2014), and intra-nigral (IN) micro-injection (Flora et al., 2003; Theodore et al., 2006a,b; Liu et al., 2014) in rats, and intra-hippocampal (IH) injection in mice (Flora et al., 2003; Theodore et al., 2006a,b; Liu et al., 2014)], and TNFα levels were further potentiated by IP injection with methamphetamine in all models examined in these studies (Flora et al., 2003; Theodore et al., 2006a,b; Liu et al., 2014). In addition, IL-1B expression was also increased by IH injection of Tat in mice (Flora et al., 2003; Theodore et al., 2006a,b; Liu et al., 2014) and by IN injection of Tat in rats (Flora et al., 2003; Theodore et al., 2006a,b; Liu et al., 2014), and further enhanced in both models by IP injection with methamphetamine (Flora et al., 2003; Theodore et al., 2006a,b; Liu et al., 2014). Moreover, IS administration of Tat coupled with IP co-exposure of rats to methamphetamine also augmented whole-tissue protein expression of IL-1α and MCP-1 (Flora et al., 2003; Theodore et al., 2006a,b; Liu et al., 2014). This deregulation of immune modulators was also accompanied by a concomitant downregulation of serotonin, dopamine, and dopamine metabolite levels in the brains of co-exposed animals (Maragos et al., 2002; Cass et al., 2003; Theodore et al., 2006a; Liu et al., 2014). Additionally, *in vivo* exposure of mice to either IH Tat or IP methamphetamine induced increases in oxidative stress, enhanced DNA-binding of select redox-responsive transcription factors (e.g., AP-1, CREB, and NFκB), and upregulated CAM (e.g., intercellular adhesion molecule-1; ICAM-1) expression, which were all potentiated by co-exposure (Flora et al., 2003). With respect to behavior, IN Tat or IP methamphetamine alone impaired the performance of rats in balance and locomotor tests, which were worsened in co-injected animals (Liu et al., 2014), implying molecular cooperation between Tat and amphetamines in the enhanced cognitive deterioration observed in HIV-1-infected patients who use amphetamines, thereby highlighting the need for improved interventions and treatment options.

### Tat and ethanol

The pleiotropic effects of ethanol have long been appreciated in the clinic and also reported in *in vitro* and animal model studies. The multifaceted consequences of ethanol exposure have also been extensively examine in the CNS, where ethanol has been shown to have multiple (sometimes polarizing) effects on a number of neurotransmitter receptors and their respective pathways, including but not limited to aberrant modulation of γ-aminobutyric acid type A (GABA-A) receptors and N-methyl-D-aspartate (NMDA) receptors in the brain (Davies, 2003; Paul, 2006). The role of co-exposure to exogenous Tat protein and ethanol on CNS cell populations cultured *in vitro* and further examined *in vivo* infer adverse consequences for alcohol-dependent HIV-1-infected patients.

*In vitro* experimentation involving exposure of primary hBMEC to exogenously applied Tat showed elevated secretion of TNFα from, and substantially increased apoptosis of, this primary cell population (Acheampong et al., 2002) and these effects were potentiated by co-exposure of hBMEC to ethanol (Acheampong et al., 2002; Brailoiu et al., 2006). In addition, Tat also enhanced intracellular calcium accumulation and apoptosis in exposed primary rat cortical neurons, which was further augmented in the presence of both compounds (Acheampong et al., 2002; Brailoiu et al., 2006), demonstrating profound cytotoxic effects of Tat on CNS cells that were accentuated in the presence of ethanol.

*Ex vivo* mono- and co-exposure of primary rat hippocampal explants to Tat and ethanol provided additional evidence of Tat-mediated toxicity being augmented in the presence of ethanol (Self et al., 2004), suggesting a tangible role for the interaction of Tat and ethanol in the development of HAND in HIV-1-infected patients; this was further corroborated by behavioral reports of *in vivo* studies wherein rats chronically exposed to ethanol (involving oral dosing three times a day for four consecutive days) demonstrated significant withdrawal-associated symptoms, which were potentiated by IH injection with Tat (Self et al., 2009). Moreover, co-exposed animals exhibited sustained decreases in task performance in water maze testing as compared to mono-exposed and control animals (Self et al., 2009), indicating more severe cognitive decline in the presence of Tat and exposure to ethanol. These behavioral results in well characterized animal models were further supported by clinical observations demonstrating more profound impairment in HIV-1-infected patients with a history of alcohol abuse as compared to HIV-1-positive non-alcohol-dependent or HIV-1-negative participants, regardless of alcohol abuse history (Green et al., 2004).

Biochemically, *in vivo* co-exposure of mice to IP injection of ethanol or IH injection of Tat resulted in elevation of ICAM-1 mRNA expression in brain tissue (Flora et al., 2005). In addition, exposure to either ethanol or Tat individually triggered increases in oxidative stress and proinflammatory cytokines (IL-1B, MCP-1, and TNFα), that were augmented in the hippocampus and corpus striatum of co-exposed mice (Flora et al., 2005), demonstrating an amplification of Tat-mediated immune deregulation in the brain by ethanol. Tat-transgenic mice (in which Tat was constitutively expressed under control of the simian virus 40 promoter) also exhibited significant defects in innate immune function (as compared to non-transgenic animals), and these were further exacerbated by either chronic oral daily dosing with ethanol for 7 days (Prakash et al., 1998a,b) or by acute dosing of one IP injection with ethanol (Prakash et al., 1998a,b). Moreover, chronic exposure of Tat-transgenic mice to ethanol and/or the drug azidothymidine (AZT) diminished *in vivo* hematopoiesis, as compared to non-exposed animals (Prakash et al., 2001), implying enhanced toxicity of cells of the hematopoietic system co-exposed to Tat, ethanol, and/or AZT.

### Tat and cannabinoids

The endocannabinoid system is modulated by the expression of two primary cannabinoid receptors, termed CB1 and CB2, which demonstrate differential enriched expression in different tissues, with CB1 receptors expressed primarily in the CNS and CB2 receptors expressed mostly on various immune cells (Pertwee, 2006; Mackie, 2008). In contrast to the predominantly negative outcomes of most recreational drug use, there is evidence to suggest a positive role for the use of cannabinoids (or simply the activation of cannabinoid receptors), particularly within the context of HIV-1 infection (Purohit et al., 2014).

Reports involving the use of *in vitro* experimental systems have demonstrated the effects of co-exposure of cannabinoids with HIV-1 Tat protein in cells relevant to the CNS. Exogenous Tat exposure of the U-937 human monocyte cell line (which express CB2, but not CB1) enhanced *in vitro* transmigration of these cells across a porous polycarbonate transwell insert (Raborn and Cabral, 2010; Raborn et al., 2014), as well as adhesion to extracellular matrix (ECM) proteins (Raborn and Cabral, 2010; Raborn et al., 2014), while pre-treatment of the monocytic cells with cannabinoids blocked these Tat-mediated transmigration and cellular adhesion effects (Raborn and Cabral, 2010; Raborn et al., 2014). Morphologically, exposure to Tat induced U-937 cells to adopt a more adherent, activated macrophage-like phenotype, whereas co-exposure with cannabinoids inhibited this phenotypic metamorphosis, thus cells maintained a non-activated monocyte-like phenotype (Raborn et al., 2014). At the molecular level, Tat also enhanced expression of the CAM B1-integrin on U-937 cells, an effect abrogated by co-treatment with cannabinoids (Raborn et al., 2014).

Exposure of the C6 rat glial cell line (which express both CB1 and CB2) with Tat yielded an increase of reactive nitrogen species (RNS) and related enzymes, which was diminished by co-exposure with a cannabinoid mimetic (Esposito et al., 2002). In addition, Tat exposure increased GFAP mRNA and protein expression in primary human Muller glia (which also express both cannabinoid receptors), and this expression was reduced by co-exposure with endocannabinoids, *in vitro* (Krishnan and Chatterjee, 2014). Furthermore, a Tat-induced decrease in glial viability was rescued by co-exposure with either a cannabinoid mimetic (Esposito et al., 2002) or endocannabinoids (Krishnan and Chatterjee, 2014).

Immunologically, Tat-mediated upregulation of a panel of pro-inflammatory cytokines (e.g., IL-1B, IL-2, IL-8, IL-12, IL-15, IFNγ, G-CSF, M-CSF, and TNFα) in primary human Muller glia was dampened by co-exposure to endocannabinoids (Krishnan and Chatterjee, 2014); remarkably, upregulation of anti-inflammatory cytokine secretion (e.g., IL-10 and TGF-β) by Tat was concomitantly enhanced in the presence of endocannabinoids (Krishnan and Chatterjee, 2014), providing evidence that cannabinoids skew cytokine production toward an anti-inflammatory profile, and demonstrating a possible use of cannabinoids in the treatment of select pro-inflammatory conditions, including HIV-1-associated diseases such as HAND. In support of *in vitro* studies, a clinical trial aimed at determining the effects of cannabinoids in HIV-1-infected patients on ART demonstrated that treatment with cannabinoids decreased serum viral RNA levels, increased CD4+ and CD8+ T-cell counts, and stimulated weight gain in HIV-1-positive patients on protease inhibitors, implying an effective adjunctive therapeutic use of cannabinoids in HIV-1-infected patients on ART (Abrams et al., 2003).

## Art and drugs in hand

The development of more effective combinations of ART over the past decade has been instrumental in the conversion of HIV-1 infection from a rapidly progressing immunodeficiency into a chronic, manageable condition with the development and deployment of an ever-increasing number of antiretroviral therapeutic agents in most industrialized countries. ART has played a critical role in the reduction of HIV-1 viral loads, boosting of CD4+ T-cell counts, and wide-ranging mitigation of the most severe symptomology associated with many of the comorbidities associated with HIV/AIDS in the earliest era of the pandemic (Wang et al., 2006; Gresele et al., 2014). These benefits were emphasized by early clinical reports of declines in incidences and severity of neurocognitive deficits and disturbances in HIV-1-infected patients using more effective ART regimens, as compared to patients on mono-therapy or no therapy (Ferrando et al., 1998; Price et al., 1999; Tozzi et al., 1999; Cohen et al., 2001). Subsequent analyses, however, comparing clinical data before and after the implementation of more effective combination therapeutic strategies have clearly demonstrated that although the overall severity of HAND exhibited in patients has declined with the introduction of combination therapeutic approaches (with a skewing of HAND symptoms to the less severe end of the spectrum) the incidence of HAND has not decreased (Cysique et al., 2004; Robertson et al., 2007; Tozzi et al., 2007; Heaton et al., 2010, 2011; Simioni et al., 2010; Cysique and Brew, 2011).

Arguably, the benefits of viral load reduction and improvement of the general health state of HIV-1-infected patients comes at the cost of toxic side-effects characteristic of current ART options (Meeker et al., 2014). Surprisingly, some HIV-1-infected patients prescribed ART regimens with a greater CNS penetration effectiveness (CPE) ranking exhibited more impaired performance on neurocognitive assessments, as compared to patients on regimens with a poorer CPE ranking, despite improved suppression of viral replication in the CNS compartment (Marra et al., 2009). In contrast, patients in a different cohort on ART with high CPE ranking demonstrated improved neurocognitive testing scores, but only if their ART regimen consisted of more than three drugs overall (Smurzynski et al., 2011). Interestingly, clinical data demonstrate progressive and sustained improvements in performance on neurocognitive assessments in HIV-1-infected patients who voluntarily discontinued long-term ART (Robertson et al., 2010). These results were observed even with continued HIV-1 viral RNA and protein production (including Tat), persistent aberrant immune activation, and enhanced amyloidosis in patients whose HIV-1 infection was considered successfully managed on ART (Falkensammer et al., 2007; Clifford et al., 2009; Yukl et al., 2009; Giunta et al., 2011; Heaton et al., 2011; Mediouni et al., 2012; Gresele et al., 2014; Smith et al., 2014), underscoring the demand for more comprehensive, improved, and adjunctive therapies in HIV-1-infected patients.

An important clinical consideration with regard to therapy regimens, regardless of toxicity, is the potential for non-adherence to ART, particularly in drug-abusing HIV-1-infected patients, who demonstrate higher non-adherence as compared to non-drug-abusing cohorts (Kamarulzaman and Altice, 2015; Kumar et al., 2015). This observation calls for the development of additional intervention strategies in these higher-risk populations, such as methadone maintenance therapy in opioid abusers, which has demonstrated promise in an increase in adherence compliance in these difficult-to-monitor populations (Lappalainen et al., 2015). Further confounding the HIV-1-infected patient treatment paradigm is the potential for pharmacological interactions between ART and various recreational drugs, as well as other prescription drugs or over-the-counter drugs; interactions which are often unrecognized, but commonly require dosing adjustments (Kumar et al., 2015; Stolbach et al., 2015).

The limitations of current ART to eliminate HIV-1 from patients despite long-term therapy or early therapeutic intervention also highlights the importance of HIV-1 reservoirs that remain unaffected by even the most rigorous therapeutic regimens. The recently reported viral rebounds of the so-called “Mississippi Baby” (Persaud et al., 2013; NIAID, 2014; Rainwater-Lovett et al., 2015), the “Boston Patients” (Henrich et al., 2013), and the VISCONTI cohort (Sáez-Cirión et al., 2013) demonstrate an ineffectiveness of currently available ART to penetrate and minimize or possibly eliminate HIV-1 reservoirs, which some speculate to include a number of resident CNS cell populations, including but not limited to perivascular macrophages, microglia, and astrocytes (Liner et al., 2010; Archin et al., 2014; Gray et al., 2014; Fois and Brew, 2015; Joseph et al., 2015).

## Conclusions

**Figure 2:**
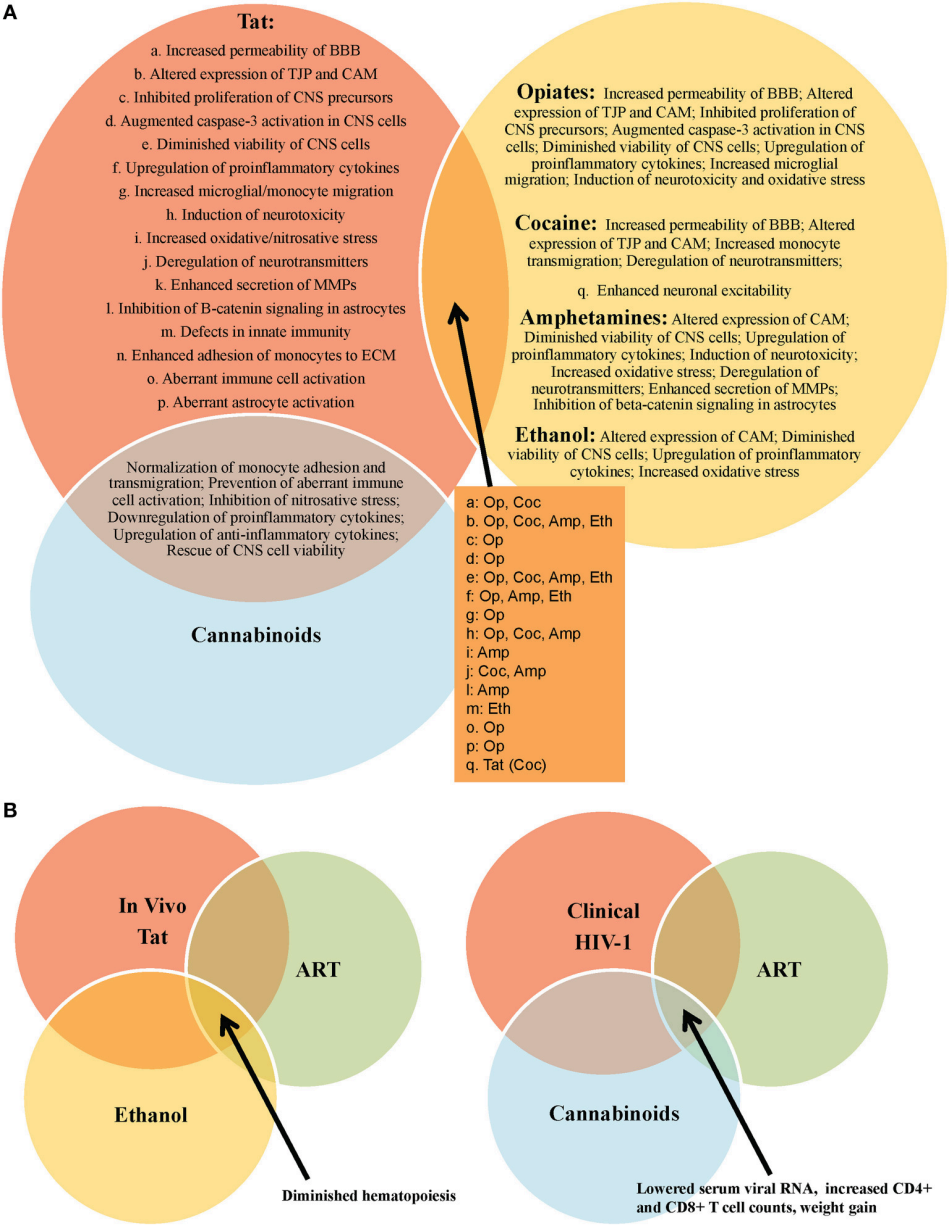
**Outcomes of HIV-1 Tat interactions with various recreational drugs and antiretroviral therapy (ART)**. **(A)** Tat or drug use alone are capable of mediating significant damage to the CNS, however, these effects are often exacerbated in the context of comorbid illicit drug use in HIV-1-infected patients. *In vitro* experiments have provided vital information on the multiple molecular-, cellular-, and tissue-altering effects of Tat in the absence and presence of drugs of abuse, including opiates (op), cocaine (coc), amphetamines (amp), and ethanol (eth). One notable exception appears to be in the case of cannabinoids, which appear to mitigate several of the negative consequences of Tat exposure, *in vitro*. **(B)** *In vivo* experiments further support *in vitro* data on the negative impact of drug use (e.g., ethanol) in the context of HIV-1 infection in patients on ART. Interestingly, a clinical report demonstrates a possible role for cannabinoids as an adjunctive therapy in HIV-1-infected patients on ART.

HIV-1 Tat mediates several detrimental consequences to multiple CNS cell populations, both *in vitro* and *in vivo*, including neurotoxicity, aberrant cellular activation, and endothelial dysfunction (Figure 1). These pathologies have been anticipated based on the spectrum of observed HAND manifested in HIV-1-infected patients (Figure 2). Clinically, Tat mRNA and protein has been consistently found in the brains of HIV-1 encephalitis (HIVE) patients, where it has been linked to a number of types of neurological impairment, including dementia (Hofman et al., 1994; Hudson et al., 2000; Chang et al., 2011). Further confounding these pathologies, genetic signatures identified within and between HIV-1 subtypes demonstrate specific phenotypic features associated with the incidence and severity of HAND, including molecular and functional compromise of the BBB (Spira et al., 2003; McArthur, 2004; Liner et al., 2007; Li et al., 2012; Bertrand et al., 2013; Dahiya et al., 2013). In addition, investigations by multiple groups have demonstrated that HIV-1 Tat is indeed produced despite clinically successful ART in well-controlled HIV-1-infected patients (Falkensammer et al., 2007; Mediouni et al., 2012). Given these observations and the fact that Tat-exposed animals exhibit many of the neurological characteristics associated with HIV-1-infected patients, these studies emphasize the need for improved therapies aimed at the direct neutralization of Tat and/or the mitigation of Tat-mediated effects in HIV-1-infected patients. These may include small molecule inhibitors of Tat, which show promise in recent *in vitro* and *in vivo* studies, demonstrating an ability to inhibit Tat-dependent transcription (Mousseau et al., 2012), as well as HIV-1 reactivation from latency (Mousseau et al., 2015), with the ability to cross the BBB and prevent inflammation in the brains of Tat-transgenic mice (Mediouni et al., 2015).

Drugs of abuse are their own confounding factor in neurological impairment and decline, and the prevalence of recreational drug use in the HIV-1-infected population continues to present obstacles for greater clinical improvements in these patients (Nath et al., 2002; Green et al., 2004; Sharma and Ali, 2006; Theodore et al., 2007; Silverstein et al., 2011; Hauser et al., 2012; Nair and Samikkannu, 2012; Smith et al., 2014; Allain et al., 2015). Moreover, recreational drug and alcohol use in HIV-1-infected patients, as well as the use of prescription medications, over-the-counter drugs, and homeopathic regimens for comorbid conditions, present the potential for dangerous pharmacological interactions in this patient population (Stolbach et al., 2015). Given these observations, there is a need for more rigorous assessments of drug use in subsets of HIV-1-infected patient populations by performing more robust drug screenings and to question the current paradigm in order to truly appreciate the impact that single- and poly-drug use really has on HIV-1 infection in general (Parikh et al., 2012, 2014). This would also necessitate an understanding of the combined effects of alcohol and tobacco in combination with any of the drugs previously discussed, and will also increasingly need to include knowledge and understanding of cannabinoids in their various formulations as the growing legalization of this drug and its use in pain management in the HIV-1-infected population is rising and will likely become more prevalently used in time. In addition, experimental and clinical Tat-mediated behavioral and molecular effects are exacerbated in the presence of a number of drugs of abuse, including opiates, cocaine, amphetamines, and ethanol (Figure 2), underscoring the additional need for withdrawal interventions and counseling in a large percentage of HIV-1-infected patients. Nonetheless, the true impact of treatments used for these drugs [including withdrawal effects both acutely (prior to the next “hit”) and chronically (during long-term suppressive therapy or by complete elimination of the drug(s))] has not been well studied. Thus, withdrawal may have additional acute and chronic effects on HIV-1 infection and associated disease with one potential consequence being reactivation of virus from reservoirs.

The development of ART has been critical in mitigating symptomatic decline and improving the overall clinical condition of those infected with HIV-1, as compared to the era before effective combination therapeutic control was available. However, the inability of current ART suppression to eliminate HIV-1 reservoirs, including those in the CNS, underscores the need for additional research into alternative strategies. In this vein, recent *in vitro* findings utilizing gene-editing technology to target and excise integrated HIV-1 proviral genomes from latently infected cells exhibit exciting potential for the development of the next generation of antiretroviral therapy on the horizon (Hu et al., 2014). Indeed, the benefits of ART are limited in the context of HAND progression in well-suppressed patients, and several reports emphasize the need for improved therapeutic alternatives in the current era of the HIV-1 pandemic (Cysique et al., 2004; Robertson et al., 2007, 2010; Tozzi et al., 2007; Marra et al., 2009; Heaton et al., 2010, 2011; Simioni et al., 2010; Cysique and Brew, 2011; Smurzynski et al., 2011). Thus, the advantages and disadvantages of ART with regard to neurocognitive impairment are currently under debate in the field and are yet to be fully determined.

## Author contributions

MM assisted in the conception and design and performed the primary writing for the review as well as Figure 1. VP and NR assisted in conception, design, and revisions of the manuscript and development of Figure 2. BW assisted in conception, design, and revisions of the manuscript. MN assisted in conception, design, and revisions of the manuscript. All authors have given final approval of the version to be published and agree to be accountable for all aspects of the work in ensuring that questions related to the accuracy or integrity of any part of the work are appropriately investigated and resolved.

## Funding

These studies were funded in part by the Public Health Service, National Institutes of Health, through grants from the National Institute of Neurological Disorders and Stroke, NS32092 and NS46263, the National Institute of Drug Abuse, DA19807 (Dr. Brian Wigdahl, Principal Investigator), National Institute of Mental Health Comprehensive NeuroAIDS Core Center (CNAC), P30 MH-092177 (Kamel Khalili, PI; BW, PI of the Drexel subcontract), and under the Ruth L. Kirschstein National Research Service Award 5T32MH079785 (Jay Rappaport, PI, BW, PI of the Drexel subcontract). The contents of the paper are solely the responsibility of the authors and do not necessarily represent the official views of the NIH. Dr. MN was supported in part by the Public Health Service, National Institutes of Health, through grants from the National Institute of Neurological Disorders and Stroke, NS089435 and faculty development funds provided by the Department of Microbiology and Immunology and the Institute for Molecular Medicine and Infectious Disease.

### Conflict of interest statement

The authors declare that the research was conducted in the absence of any commercial or financial relationships that could be construed as a potential conflict of interest.

## References

[B1] Abrams D. I. Hilton J. F. Leiser R. J. Shade S. B. Elbeik T. A. Aweeka F. T.. (2003). Short-term effects of cannabinoids in patients with HIV-1 infection: a randomized, placebo-controlled clinical trial. Ann. Intern. Med. 139, 258–266. 10.7326/0003-4819-139-4-200308190-00008 12965981

[B2] Acheampong E. Mukhtar M. Parveen Z. Ngoubilly N. Ahmad N. Patel C.. (2002). Ethanol strongly potentiates apoptosis induced by HIV-1 proteins in primary human brain microvascular endothelial cells. Virology 304, 222–234. 10.1006/viro.2002.1666 12504564

[B3] Aksenov M. Y. Aksenova M. V. Nath A. Ray P. D. Mactutus C. F. Booze R. M. (2006). Cocaine-mediated enhancement of Tat toxicity in rat hippocampal cell cultures: the role of oxidative stress and D1 dopamine receptor. Neurotoxicology 27, 217–228. 10.1016/j.neuro.2005.10.003 16386305

[B4] Al-Hasani R. Bruchas M. R. (2011). Molecular mechanisms of opioid receptor-dependent signaling and behavior. Anesthesiology 115, 1363–1381. 10.1097/aln.0b013e318238bba6 22020140 PMC3698859

[B5] Allain F. Minogianis E. A. Roberts D. C. Samaha A. N. (2015). How fast and how often: the pharmacokinetics of drug use are decisive in addiction. Neurosci. Biobehav. Rev. 56, 166–179. 10.1016/j.neubiorev.2015.06.012 26116543

[B6] András I. E. Pu H. Deli M. A. Nath A. Hennig B. Toborek M. (2003). HIV-1 Tat protein alters tight junction protein expression and distribution in cultured brain endothelial cells. J. Neurosci. Res. 74, 255–265. 10.1002/jnr.10762 14515355

[B7] Antinori A. Arendt G. Becker J. T. Brew B. J. Byrd D. A. Cherner M.. (2007). Updated research nosology for HIV-associated neurocognitive disorders. Neurology 69, 1789–1799. 10.1212/01.WNL.0000287431.88658.8b 17914061 PMC4472366

[B8] Aprea S. Del Valle L. Mameli G. Sawaya B. E. Khalili K. Peruzzi F. (2006). Tubulin-mediated binding of human immunodeficiency virus-1 Tat to the cytoskeleton causes proteasomal-dependent degradation of microtubule-associated protein 2 and neuronal damage. J. Neurosci. 26, 4054–4062. 10.1523/JNEUROSCI.0603-06.2006 16611822 PMC6673895

[B9] Archin N. M. Sung J. M. Garrido C. Soriano-Sarabia N. Margolis D. M. (2014). Eradicating HIV-1 infection: seeking to clear a persistent pathogen. Nat. Rev. Microbiol. 12, 750–764. 10.1038/nrmicro3352 25402363 PMC4383747

[B10] Bagashev A. Sawaya B. E. (2013). Roles and functions of HIV-1 Tat protein in the CNS: an overview. Virol. J. 10:358. 10.1186/1743-422X-10-358 24359561 PMC3879180

[B11] Bell J. (2014). Pharmacological maintenance treatments of opiate addiction. Br. J. Clin. Pharmacol. 77, 253–263. 10.1111/bcp.12051 23210630 PMC4014029

[B12] Bertrand S. J. Aksenova M. V. Mactutus C. F. Booze R. M. (2013). HIV-1 Tat protein variants: critical role for the cysteine region in synaptodendritic injury. Exp. Neurol. 248, 228–235. 10.1016/j.expneurol.2013.06.020 23811015 PMC3773475

[B13] Blaylock B. L. Nader M. A. (2012). Dopamine D3 receptor function and cocaine exposure. Neuropsychopharmacology 37, 297–298. 10.1038/npp.2011.170 22157862 PMC3238064

[B14] Bokhari S. M. Yao H. Bethel-Brown C. Fuwang P. Williams R. Dhillon N. K.. (2009). Morphine enhances Tat-induced activation in murine microglia. J. Neurovirol. 15, 219–228. 10.1080/13550280902913628 19462331 PMC3121575

[B15] Brailoiu E. Brailoiu G. C. Mameli G. Dolei A. Sawaya B. E. Dun N. J. (2006). Acute exposure to ethanol potentiates human immunodeficiency virus type 1 Tat-induced Ca(2+) overload and neuronal death in cultured rat cortical neurons. J. Neurovirol. 12, 17–24. 10.1080/13550280500516427 16595370

[B16] Bruce-Keller A. J. Chauhan A. Dimayuga F. O. Gee J. Keller J. N. Nath A. (2003). Synaptic transport of human immunodeficiency virus-Tat protein causes neurotoxicity and gliosis in rat brain. J. Neurosci. 23, 8417–8422. 12968004 10.1523/JNEUROSCI.23-23-08417.2003 PMC6740701

[B17] Bruce-Keller A. J. Turchan-Cholewo J. Smart E. J. Geurin T. Chauhan A. Reid R.. (2008). Morphine causes rapid increases in glial activation and neuronal injury in the striatum of inducible HIV-1 Tat transgenic mice. Glia 56, 1414–1427. 10.1002/glia.20708 18551626 PMC2725184

[B18] Buch S. K. Khurdayan V. K. Lutz S. E. Knapp P. E. El-Hage N. Hauser K. F. (2007). Glial-restricted precursors: patterns of expression of opioid receptors and relationship to human immunodeficiency virus-1 Tat and morphine susceptibility *in vitro*. Neuroscience 146, 1546–1554. 10.1016/j.neuroscience.2007.03.006 17478053 PMC4308314

[B19] Calipari E. S. Ferris M. J. (2013). Amphetamine mechanisms and actions at the dopamine terminal revisited. J. Neurosci. 33, 8923–8925. 10.1523/JNEUROSCI.1033-13.2013 23699503 PMC3753078

[B20] Cass W. A. Harned M. E. Peters L. E. Nath A. Maragos W. F. (2003). HIV-1 protein Tat potentiation of methamphetamine-induced decreases in evoked overflow of dopamine in the striatum of the rat. Brain Res. 984, 133–142. 10.1016/S0006-8993(03)03122-6 12932847

[B21] Chang H. C. Samaniego F. Nair B. C. Buonaguro L. Ensoli B. (1997). HIV-1 Tat protein exits from cells via a leaderless secretory pathway and binds to extracellular matrix-associated heparan sulfate proteoglycans through its basic region. AIDS 11, 1421–1431. 10.1097/00002030-199712000-00006 9342064

[B22] Chang J. R. Mukerjee R. Bagashev A. Del Valle L. Chabrashvili T. Hawkins B. J.. (2011). HIV-1 Tat protein promotes neuronal dysfunction through disruption of microRNAs. J. Biol. Chem. 286, 41125–41134. 10.1074/jbc.M111.268466 21956116 PMC3220514

[B23] Chang S. L. Connaghan K. P. Wei Y. Li M. D. (2014). NeuroHIV and use of addictive substances. Int. Rev. Neurobiol. 118, 403–440. 10.1016/B978-0-12-801284-0.00013-0 25175871

[B24] Chauhan A. Turchan J. Pocernich C. Bruce-Keller A. Roth S. Butterfield D. A.. (2003). Intracellular human immunodeficiency virus Tat expression in astrocytes promotes astrocyte survival but induces potent neurotoxicity at distant sites via axonal transport. J. Biol. Chem. 278, 13512–13519. 10.1074/jbc.M209381200 12551932

[B25] Clifford D. B. Fagan A. M. Holtzman D. M. Morris J. C. Teshome M. Shah A. R.. (2009). CSF biomarkers of Alzheimer disease in HIV-associated neurologic disease. Neurology 73, 1982–1987. 10.1212/WNL.0b013e3181c5b445 19907013 PMC2790234

[B26] Cohen R. A. Boland R. Paul R. Tashima K. T. Schoenbaum E. E. Celentano D. D.. (2001). Neurocognitive performance enhanced by highly active antiretroviral therapy in HIV-infected women. AIDS 15, 341–345. 10.1097/00002030-200102160-00007 11273214

[B27] Conant K. St Hillaire C. Anderson C. Galey D. Wang J. Nath A. (2004). Human immunodeficiency virus type 1 Tat and methamphetamine affect the release and activation of matrix-degrading proteinases. J. Neurovirol. 10, 21–28. 10.1080/13550280490261699 14982725

[B28] Cysique L. A. Brew B. J. (2011). Prevalence of non-confounded HIV-associated neurocognitive impairment in the context of plasma HIV RNA suppression. J. Neurovirol. 17, 176–183. 10.1007/s13365-011-0021-x 21416169

[B29] Cysique L. A. Maruff P. Brew B. J. (2004). Prevalence and pattern of neuropsychological impairment in human immunodeficiency virus-infected/acquired immunodeficiency syndrome (HIV/AIDS) patients across pre- and post-highly active antiretroviral therapy eras: a combined study of two cohorts. J. Neurovirol. 10, 350–357. 10.1080/13550280490521078 15765806

[B30] Dahiya S. Irish B. P. Nonnemacher M. R. Wigdahl B. (2013). Genetic variation and HIV-associated neurologic disease. Adv. Virus Res. 87, 183–240. 10.1016/B978-0-12-407698-3.00006-5 23809924 PMC4074542

[B31] Davies M. (2003). The role of GABAA receptors in mediating the effects of alcohol in the central nervous system. J. Psychiatry Neurosci. 28, 263–274. 12921221 PMC165791

[B32] Debaisieux S. Rayne F. Yezid H. Beaumelle B. (2012). The ins and outs of HIV-1 Tat. Traffic 13, 355–363. 10.1111/j.1600-0854.2011.01286.x 21951552

[B33] El-Hage N. Bruce-Keller A. J. Yakovleva T. Bazov I. Bakalkin G. Knapp P. E.. (2008). Morphine exacerbates HIV-1 Tat-induced cytokine production in astrocytes through convergent effects on [Ca(2+)](i), NF-kappaB trafficking and transcription. PLoS ONE 3:e4093. 10.1371/journal.pone.0004093 19116667 PMC2605563

[B34] El-Hage N. Gurwell J. A. Singh I. N. Knapp P. E. Nath A. Hauser K. F. (2005). Synergistic increases in intracellular Ca2+, and the release of MCP-1, RANTES, and IL-6 by astrocytes treated with opiates and HIV-1 Tat. Glia 50, 91–106. 10.1002/glia.20148 15630704 PMC4301446

[B35] El-Hage N. Wu G. Wang J. Ambati J. Knapp P. E. Reed J. L.. (2006). HIV-1 Tat and opiate-induced changes in astrocytes promote chemotaxis of microglia through the expression of MCP-1 and alternative chemokines. Glia 53, 132–146. 10.1002/glia.20262 16206161 PMC3077280

[B36] Ensoli B. Buonaguro L. Barillari G. Fiorelli V. Gendelman R. Morgan R. A.. (1993). Release, uptake, and effects of extracellular human immunodeficiency virus type 1 Tat protein on cell growth and viral transactivation. J. Virol. 67, 277–287. 8416373 10.1128/jvi.67.1.277-287.1993 PMC237361

[B37] Esposito G. Ligresti A. Izzo A. A. Bisogno T. Ruvo M. Di Rosa M.. (2002). The endocannabinoid system protects rat glioma cells against HIV-1 Tat protein-induced cytotoxicity. Mechan. Regul. 277, 50348–50354. 10.1074/jbc.M207170200 12388547

[B38] Falkensammer B. Freissmuth D. Hübner L. Speth C. Dierich M. P. Stoiber H. (2007). Changes in HIV-specific antibody responses and neutralization titers in patients under ART. Front. Biosci. 12, 2148–2158. 10.2741/2218 17127452

[B39] Feng Y. He X. Yang Y. Chao D. Lazarus L. H. Xia Y. (2012). Current research on opioid receptor function. Curr. Drug Targets 13, 230–246. 10.2174/138945012799201612 22204322 PMC3371376

[B40] Ferrando S. van Gorp W. McElhiney M. Goggin K. Sewell M. Rabkin J. (1998). Highly active antiretroviral treatment in HIV infection: benefits for neuropsychological function. AIDS 12, F65–F70. 10.1097/00002030-199808000-00002 9631133

[B41] Ferris M. J. Frederick-Duus D. Fadel J. Mactutus C. F. Booze R. M. (2010). Hyperdopaminergic tone in HIV-1 protein treated rats and cocaine sensitization. J. Neurochem. 115, 885–896. 10.1111/j.1471-4159.2010.06968.x 20796175 PMC4041991

[B42] Fitting S. Knapp P. E. Zou S. Marks W. D. Bowers M. S. Akbarali H. I.. (2014). Interactive HIV-1 Tat and morphine-induced synaptodendritic injury is triggered through focal disruptions in Na(+) influx, mitochondrial instability, and Ca(2)(+) overload. J. Neurosci. 34, 12850–12864. 10.1523/JNEUROSCI.5351-13.2014 25232120 PMC4166164

[B43] Fitting S. Scoggins K. L. Xu R. Dever S. M. Knapp P. E. Dewey W. L.. (2012). Morphine efficacy is altered in conditional HIV-1 Tat transgenic mice. Eur. J. Pharmacol. 689, 96–103. 10.1016/j.ejphar.2012.05.029 22659585 PMC3402587

[B44] Flora G. Lee Y. W. Nath A. Hennig B. Maragos W. Toborek M. (2003). Methamphetamine potentiates HIV-1 Tat protein-mediated activation of redox-sensitive pathways in discrete regions of the brain. Exp. Neurol. 179, 60–70. 10.1006/exnr.2002.8048 12504868

[B45] Flora G. Pu H. Lee Y. W. Ravikumar R. Nath A. Hennig B.. (2005). Proinflammatory synergism of ethanol and HIV-1 Tat protein in brain tissue. Exp. Neurol. 191, 2–12. 10.1016/j.expneurol.2004.06.007 15589507

[B46] Fois A. F. Brew B. J. (2015). The potential of the CNS as a reservoir for HIV-1 infection: implications for HIV eradication. Curr. HIV/AIDS Rep. 12, 299–303. 10.1007/s11904-015-0257-9 25869939

[B47] Frankel A. D. Young J. A. (1998). HIV-1: fifteen proteins and an RNA. Annu. Rev. Biochem. 67, 1–25. 10.1146/annurev.biochem.67.1.1 9759480

[B48] Friedrich B. M. Dziuba N. Li G. Endsley M. A. Murray J. L. Ferguson M. R. (2011). Host factors mediating HIV-1 replication. Virus Res. 161, 101–114. 10.1016/j.virusres.2011.08.001 21871504

[B49] Gandhi N. Saiyed Z. M. Napuri J. Samikkannu T. Reddy P. V. Agudelo M.. (2010). Interactive role of human immunodeficiency virus type 1 (HIV-1) clade-specific Tat protein and cocaine in blood-brain barrier dysfunction: implications for HIV-1-associated neurocognitive disorder. J. Neurovirol. 16, 294–305. 10.3109/13550284.2010.499891 20624003

[B50] Giunta B. Ehrhart J. Obregon D. F. Lam L. Le L. Jin J.. (2011). Antiretroviral medications disrupt microglial phagocytosis of beta-amyloid and increase its production by neurons: implications for HIV-associated neurocognitive disorders. Mol. Brain 4, 23. 10.1186/1756-6606-4-23 21649911 PMC3128056

[B51] Gray L. R. Roche M. Flynn J. K. Wesselingh S. L. Gorry P. R. Churchill M. J. (2014). Is the central nervous system a reservoir of HIV-1? Curr. Opin. HIV AIDS 9, 552–558. 10.1097/COH.0000000000000108 25203642 PMC4215931

[B52] Green J. E. Saveanu R. V. Bornstein R. A. (2004). The effect of previous alcohol abuse on cognitive function in HIV infection. Am. J. Psychiatry 161, 249–254. 10.1176/appi.ajp.161.2.249 14754773

[B53] Gresele P. Falcinelli E. Momi S. Francisci D. Baldelli F. (2014). Highly active antiretroviral therapy-related mechanisms of endothelial and platelet function alterations. Rev. Cardiovasc. Med. 15 (Suppl.1), S9–S20. 24987863

[B54] Hahn Y. K. Podhaizer E. M. Hauser K. F. Knapp P. E. (2012). HIV-1 alters neural and glial progenitor cell dynamics in the central nervous system: coordinated response to opiates during maturation. Glia 60, 1871–1887. 10.1002/glia.22403 22865725 PMC4030306

[B55] Harrod S. B. Mactutus C. F. Fitting S. Hasselrot U. Booze R. M. (2008). Intra-accumbal Tat1-72 alters acute and sensitized responses to cocaine. Pharmacol. Biochem. Behav. 90, 723–729. 10.1016/j.pbb.2008.05.020 18582493 PMC2703478

[B56] Hauser K. F. Fitting S. Dever S. M. Podhaizer E. M. Knapp P. E. (2012). Opiate drug use and the pathophysiology of neuroAIDS. Curr. HIV Res. 10, 435–452. 10.2174/157016212802138779 22591368 PMC3431547

[B57] Hauser K. F. Hahn Y. K. Adjan V. V. Zou S. Buch S. K. Nath A.. (2009). HIV-1 Tat and morphine have interactive effects on oligodendrocyte survival and morphology. Glia 57, 194–206. 10.1002/glia.20746 18756534 PMC2743138

[B58] Heaton R. K. Clifford D. B. Franklin D. R. Jr. Woods S. P. Ake C. Vaida F.. (2010). HIV-associated neurocognitive disorders persist in the era of potent antiretroviral therapy: CHARTER Study. Neurology 75, 2087–2096. 10.1212/WNL.0b013e318200d727 21135382 PMC2995535

[B59] Heaton R. K. Franklin D. R. Ellis R. J. McCutchan J. A. Letendre S. L. Leblanc S.. (2011). HIV-associated neurocognitive disorders before and during the era of combination antiretroviral therapy: differences in rates, nature, and predictors. J. Neurovirol. 17, 3–16. 10.1007/s13365-010-0006-1 21174240 PMC3032197

[B60] Henrich T. J. Hu Z. Li J. Z. Sciaranghella G. Busch M. P. Keating S. M.. (2013). Long-term reduction in peripheral blood HIV type 1 reservoirs following reduced-intensity conditioning allogeneic stem cell transplantation. J. Infect. Dis. 207, 1694–1702. 10.1093/infdis/jit086 23460751 PMC3636784

[B61] Hofman F. M. Dohadwala M. M. Wright A. D. Hinton D. R. Walker S. M. (1994). Exogenous tat protein activates central nervous system-derived endothelial cells. J. Neuroimmunol. 54, 19–28. 10.1016/0165-5728(94)90226-7 7523444

[B62] Holtz T. H. Pattanasin S. Chonwattana W. Tongtoyai J. Chaikummao S. Varangrat A.. (2015). Longitudinal analysis of key HIV-risk behavior patterns and predictors in men who have sex with men, Bangkok, Thailand. Arch. Sex. Behav. 44, 341–348. 10.1007/s10508-014-0427-7 25637308

[B63] Hu W. Kaminski R. Yang F. Zhang Y. Cosentino L. Li F.. (2014). RNA-directed gene editing specifically eradicates latent and prevents new HIV-1 infection. Proc. Natl. Acad. Sci. U.S.A. 111, 11461–11466. 10.1073/pnas.1405186111 25049410 PMC4128125

[B64] Huang Y. F. Yang J. Y. Nelson K. E. Kuo H. S. Lew-Ting C. Y. Yang C. H.. (2014). Changes in HIV incidence among people who inject drugs in Taiwan following introduction of a harm reduction program: a study of two cohorts. PLoS Med. 11:e1001625. 10.1371/journal.pmed.1001625 24714449 PMC3979649

[B65] Hudson L. Liu J. Nath A. Jones M. Raghavan R. Narayan O.. (2000). Detection of the human immunodeficiency virus regulatory protein tat in CNS tissues. J. Neurovirol. 6, 145–155. 10.3109/13550280009013158 10822328

[B66] Jeang K. T. Xiao H. Rich E. A. (1999). Multifaceted activities of the HIV-1 transactivator of transcription, Tat. J. Biol. Chem. 274, 28837–28840. 10.1074/jbc.274.41.28837 10506122

[B67] Joseph S. B. Arrildt K. T. Sturdevant C. B. Swanstrom R. (2015). HIV-1 target cells in the CNS. J. Neurovirol. 21, 276–289. 10.1007/s13365-014-0287-x 25236812 PMC4366351

[B68] Kalantari P. Harandi O. F. Hankey P. A. Henderson A. J. (2008). HIV-1 Tat mediates degradation of RON receptor tyrosine kinase, a regulator of inflammation. J. Immunol. 181, 1548–1555. 10.4049/jimmunol.181.2.1548 18606710 PMC2536764

[B69] Kamarulzaman A. Altice F. L. (2015). Challenges in managing HIV in people who use drugs. Curr. Opin. Infect. Dis. 28, 10–16. 10.1097/QCO.0000000000000125 25490106 PMC4409950

[B70] Kim B. O. Liu Y. Zhou B. Y. He J. J. (2004). Induction of C chemokine XCL1 (lymphotactin/single C motif-1 alpha/activation-induced, T cell-derived and chemokine-related cytokine) expression by HIV-1 Tat protein. J. Immunol. 172, 1888–1895. 10.4049/jimmunol.172.3.1888 14734774

[B71] Kim T. A. Avraham H. K. Koh Y. H. Jiang S. Park I. W. Avraham S. (2003). HIV-1 Tat-mediated apoptosis in human brain microvascular endothelial cells. J. Immunol. 170, 2629–2637. 10.4049/jimmunol.170.5.2629 12594291

[B72] Kolson D. L. Collman R. Hrin R. Balliet J. W. Laughlin M. McGann K. A.. (1994). Human immunodeficiency virus type 1 Tat activity in human neuronal cells: uptake and trans-activation. J. Gen. Virol. 75(Pt 8), 1927–1934. 10.1099/0022-1317-75-8-1927 8046394

[B73] Krishnan G. Chatterjee N. (2014). Endocannabinoids affect innate immunity of Muller glia during HIV-1 Tat cytotoxicity. Mol. Cell. Neurosci. 59, 10–23. 10.1016/j.mcn.2014.01.001 24418364

[B74] Kumar S. Rao P. S. Earla R. Kumar A. (2015). Drug-drug interactions between anti-retroviral therapies and drugs of abuse in HIV systems. Exp. Opin. Drug Metabol. Toxicol. 11, 343–355. 10.1517/17425255.2015.996546 25539046 PMC4428551

[B75] Lappalainen L. Nolan S. Dobrer S. Puscas C. Montaner J. Ahamad K.. (2015). Dose-response relationship between methadone dose and adherence to antiretroviral therapy among HIV-positive people who use illicit opioids. Addiction 110, 1330–1339. 10.1111/add.12970 25940906 PMC4503496

[B76] Levine A. J. Reynolds S. Cox C. Miller E. N. Sinsheimer J. S. Becker J. T. (2014). Neuropsychology Working Group of the Multicenter, The longitudinal and interactive effects of HIV status, stimulant use, and host genotype upon neurocognitive functioning. J. Neurovirol. 20, 243–257. 10.1007/s13365-014-0241-y 24737013 PMC4040160

[B77] Li L. Dahiya S. Kortagere S. Aiamkitsumrit B. Cunningham D. Pirrone V.. (2012). Impact of Tat Genetic Variation on HIV-1 Disease. Adv. Virol. 2012:123605. 10.1155/2012/123605 22899925 PMC3414192

[B78] Li W. Li G. Steiner J. Nath A. (2009). Role of Tat protein in HIV neuropathogenesis. Neurotox. Res. 16, 205–220. 10.1007/s12640-009-9047-8 19526283

[B79] Liner K. J. II Hall C. D. Robertson K. R. (2007). Impact of human immunodeficiency virus (HIV) subtypes on HIV-associated neurological disease. J. Neurovirol. 13, 291–304. 10.1080/13550280701422383 17849313

[B80] Liner K. J. II Ro M. J. Robertson K. R. (2010). HIV, antiretroviral therapies, and the brain. Curr. HIV/AIDS Rep. 7, 85–91. 10.1007/s11904-010-0042-8 20425562

[B81] Liu W. Y. Wang Z. B. Zhang L. C. Wei X. Li L. (2012). Tight junction in blood-brain barrier: an overview of structure, regulation, and regulator substances. CNS Neurosci. Ther. 18, 609–615. 10.1111/j.1755-5949.2012.00340.x 22686334 PMC6493516

[B82] Liu Z. Shi Z. Liu J. Wang Y. (2014). HIV transactivator of transcription enhances methamphetamine-induced Parkinson's-like behavior in the rats. Neuroreport 25, 860–864. 10.1097/WNR.0000000000000199 PMC4236185 24911386

[B83] Ma M. Nath A. (1997). Molecular determinants for cellular uptake of Tat protein of human immunodeficiency virus type 1 in brain cells. J. Virol. 71, 2495–2499. 9032389 10.1128/jvi.71.3.2495-2499.1997 PMC191362

[B84] Mackie K. (2008). Cannabinoid receptors: where they are and what they do. J. Neuroendocrinol. 20 (Suppl. 1), 10–14. 10.1111/j.1365-2826.2008.01671.x 18426493

[B85] Mahajan S. D. Aalinkeel R. Sykes D. E. Reynolds J. L. Bindukumar B. Fernandez S. F.. (2008). Tight junction regulation by morphine and HIV-1 tat modulates blood-brain barrier permeability. J. Clin. Immunol. 28, 528–541. 10.1007/s10875-008-9208-1 18574677

[B86] Malik S. Khalique H. Buch S. Seth P. (2011). A growth factor attenuates HIV-1 Tat and morphine induced damage to human neurons: implication in HIV/AIDS-drug abuse cases. PLoS ONE 6:e18116. 10.1371/journal.pone.0018116 21483469 PMC3063804

[B87] Maragos W. F. Young K. L. Turchan J. T. Guseva M. Pauly J. R. Nath A.. (2002). Human immunodeficiency virus-1 Tat protein and methamphetamine interact synergistically to impair striatal dopaminergic function. J. Neurochem. 83, 955–963. 10.1046/j.1471-4159.2002.01212.x 12421368

[B88] Marra C. M. Zhao Y. Clifford D. B. Letendre S. Evans S. Henry K.. (2009). Impact of combination antiretroviral therapy on cerebrospinal fluid HIV RNA and neurocognitive performance. AIDS 23, 1359–1366. 10.1097/QAD.0b013e32832c4152 19424052 PMC2706549

[B89] McArthur J. C. (2004). HIV dementia: an evolving disease. J. Neuroimmunol. 157, 3–10. 10.1016/j.jneuroim.2004.08.042 15579274

[B90] Mediouni S. Darque A. Baillat G. Ravaux I. Dhiver C. Tissot-Dupont H.. (2012). Antiretroviral therapy does not block the secretion of the human immunodeficiency virus tat protein. Infect. Disord. Drug Targets 12, 81–86. 10.2174/187152612798994939 22280310

[B91] Mediouni S. Jablonski J. Paris J. J. Clementz M. A. Thenin-Houssier S. McLaughlin J. P.. (2015). Didehydro-cortistatin A inhibits HIV-1 Tat mediated neuroinflammation and prevents potentiation of cocaine reward in Tat transgenic mice. Curr. HIV Res. 13, 64–79. 10.2174/1570162X13666150121111548 25613133 PMC4416414

[B92] Meeker R. B. Asahchop E. Power C. (2014). The brain and HAART: collaborative and combative connections. Curr. Opin. HIV AIDS 9, 579–584. 10.1097/COH.0000000000000110 25275707

[B93] Mishra R. Singh S. K. (2014). HIV-1 Tat C phosphorylates VE-cadherin complex and increases human brain microvascular endothelial cell permeability. BMC Neurosci. 15:80. 10.1186/1471-2202-15-80 24965120 PMC4230799

[B94] Mousseau G. Clementz M. A. Bakeman W. N. Nagarsheth N. Cameron M. Shi J.. (2012). An analog of the natural steroidal alkaloid cortistatin A potently suppresses Tat-dependent HIV transcription. Cell Host Microbe 12, 97–108. 10.1016/j.chom.2012.05.016 22817991 PMC3403716

[B95] Mousseau G. Kessing C. F. Fromentin R. Trautmann L. Chomont N. Valente S. T. (2015). The tat inhibitor didehydro-cortistatin a prevents HIV-1 reactivation from latency. mBio 6:e00465. 10.1128/mBio.00465-15 26152583 PMC4495168

[B96] Nair M. P. Samikkannu T. (2012). Differential regulation of neurotoxin in HIV clades: role of cocaine and methamphetamine. Curr. HIV Res. 10, 429–434. 10.2174/157016212802138742 22591367

[B97] Napier T. C. Chen L. Kashanchi F. Hu X. T. (2014). Repeated cocaine treatment enhances HIV-1 Tat-induced cortical excitability via over-activation of L-type calcium channels. J. Neuroimm. Pharmacol. 9, 354–368. 10.1007/s11481-014-9524-6 24567038 PMC4019717

[B98] Nasi M. Pinti M. De Biasi S. Gibellini L. Ferraro D. Mussini C.. (2014). Aging with HIV infection: a journey to the center of inflammAIDS, immunosenescence and neuroHIV. Immunol. Lett. 162, 329–333. 10.1016/j.imlet.2014.06.012 24996041

[B99] Nath A. Hauser K. F. Wojna V. Booze R. M. Maragos W. Prendergast M.. (2002). Molecular basis for interactions of HIV and drugs of abuse. J. Acquir. Imm. Defi. Syndr. 31 (Suppl. 2), S62–S69. 10.1097/00126334-200210012-00006 12394784

[B100] Nestler E. J. (2005). The neurobiology of cocaine addiction. Sci. Pract. Perspect. 3, 4–10. 10.1151/spp05314 18552739 PMC2851032

[B101] NIAID R. (2014). “Mississippi Baby” Now has Detectable HIV, Researchers find, US Department of Health and Human Services, NIH News.

[B102] Nishijima T. Gatanaga H. Komatsu H. Takano M. Ogane M. Ikeda K.. (2013). High prevalence of illicit drug use in men who have sex with men with HIV-1 infection in Japan. PLoS ONE 8:e81960. 10.1371/journal.pone.0081960 24339982 PMC3858294

[B103] Norman J. P. Perry S. W. Kasischke K. A. Volsky D. J. Gelbard H. A. (2007). HIV-1 trans activator of transcription protein elicits mitochondrial hyperpolarization and respiratory deficit, with dysregulation of complex IV and nicotinamide adenine dinucleotide homeostasis in cortical neurons. J. Immunol. 178, 869–876. 10.4049/jimmunol.178.2.869 17202348

[B104] Parikh N. Dampier W. Feng R. Passic S. R. Zhong W. Frantz B.. (2014). Cocaine alters cytokine profiles in HIV-1-infected African American individuals in the DrexelMed HIV/AIDS genetic analysis cohort. J. Acquir. Immune. Defic. Syndr. 66, 256–264. 10.1097/QAI.0000000000000163 24732878 PMC4146625

[B105] Parikh N. Nonnemacher M. R. Pirrone V. Block T. Mehta A. Wigdahl B. (2012). Substance abuse, HIV-1 and hepatitis. Curr. HIV Res. 10, 557–571. 10.2174/157016212803306023 22973853 PMC3708479

[B106] Paris J. J. Carey A. N. Shay C. F. Gomes S. M. He J. J. McLaughlin J. P. (2014). Effects of conditional central expression of HIV-1 tat protein to potentiate cocaine-mediated psychostimulation and reward among male mice. Neuropsychopharmacology 39, 380–388. 10.1038/npp.2013.201 23945478 PMC3870789

[B107] Paul S. M. (2006). Alcohol-sensitive GABA receptors and alcohol antagonists. Proc. Natl. Acad. Sci. U.S.A. 103, 8307–8308. 10.1073/pnas.0602862103 16717187 PMC1482489

[B108] Persaud D. Gay H. Ziemniak C. Chen Y. H. Piatak M. Jr. Chun T. W.. (2013). Absence of detectable HIV-1 viremia after treatment cessation in an infant. N. Engl. J. Med. 369, 1828–1835. 10.1056/NEJMoa1302976 24152233 PMC3954754

[B109] Pertwee R. G. (2006). The pharmacology of cannabinoid receptors and their ligands: an overview. Intern. J. Obesity 30(Suppl. 1), S13–S18. 10.1038/sj.ijo.0803272 16570099

[B110] Pirrone V. Libon D. J. Sell C. Lerner C. A. Nonnemacher M. R. Wigdahl B. (2013). Impact of age on markers of HIV-1 disease. Future Virol. 8, 81–101. 10.2217/fvl.12.127 23596462 PMC3625689

[B111] Prakash O. Joshi B. H. Zhang P. Aw T. Y. Teng S. Ali M.. (1998a). Transgenic mouse model of ethanol as a cofactor in HIV disease. Alcoholism Clin. Exp. Res. 22, 266S–268S. 10.1111/j.1530-0277.1998.tb04015.x 9727649

[B112] Prakash O. Rodriguez V. E. Tang Z. Y. Zhou P. Coleman R. Dhillon G.. (2001). Inhibition of hematopoietic progenitor cell proliferation by ethanol in human immunodeficiency virus type 1 tat-expressing transgenic mice. Alcohol. Clin. Exp. Res. 25, 450–456. 10.1111/j.1530-0277.2001.tb02234.x 11290858

[B113] Prakash O. Zhang P. Xie M. Ali M. Zhou P. Coleman R.. (1998b). The human immunodeficiency virus type I Tat protein potentiates ethanol-induced neutrophil functional impairment in transgenic mice. Alcohol. Clin. Exp. Res. 22, 2043–2049. 10.1097/00000374-199812000-00021 9884149

[B114] Price R. W. Yiannoutsos C. T. Clifford D. B. Zaborski L. Tselis A. Sidtis J. J.. (1999). Neurological outcomes in late HIV infection: adverse impact of neurological impairment on survival and protective effect of antiviral therapy. AIDS Clin. Trial Group Neurol. AIDS Res. Consort. Study Team. Aids 13, 1677–1685. 10.1097/00002030-199909100-00011 10509569

[B115] Pu H. Tian J. Flora G. Lee Y. W. Nath A. Hennig B.. (2003). HIV-1 Tat protein upregulates inflammatory mediators and induces monocyte invasion into the brain. Mol. Cell. Neurosci. 24, 224–237. 10.1016/S1044-7431(03)00171-4 14550782

[B116] Purohit V. Rapaka R. S. Rutter J. (2014). Cannabinoid receptor-2 and HIV-associated neurocognitive disorders. J. Neuroimm. Pharmacol. 9, 447–453. 10.1007/s11481-014-9554-0 25015040

[B117] Qi L. Gang L. Hang K. W. Ling C. H. Xiaofeng Z. Zhen L.. (2011). Programmed neuronal cell death induced by HIV-1 tat and methamphetamine. Microsc. Res. Tech. 74, 1139–1144. 10.1002/jemt.21006 21563266

[B118] Raborn E. S. Cabral G. A. (2010). Cannabinoid inhibition of macrophage migration to the trans-activating (Tat) protein of HIV-1 is linked to the CB(2) cannabinoid receptor. J. Pharmacol. Exp. Ther. 333, 319–327. 10.1124/jpet.109.163055 20089805 PMC2846023

[B119] Raborn E. S. Jamerson M. Marciano-Cabral F. Cabral G. A. (2014). Cannabinoid inhibits HIV-1 Tat-stimulated adhesion of human monocyte-like cells to extracellular matrix proteins. Life Sci. 104, 15–23. 10.1016/j.lfs.2014.04.008 24742657 PMC4089388

[B120] Rainwater-Lovett K. Luzuriaga K. Persaud D. (2015). Very early combination antiretroviral therapy in infants: prospects for cure. Curr. Opin. HIV AIDS 10, 4–11. 10.1097/COH.0000000000000127 25402708 PMC4351817

[B121] Ramakrishnan R. Chiang K. Liu H. Budhiraja S. Donahue H. Rice A. P. (2012). Making a short story long: regulation of P-TEFb and HIV-1 transcriptional elongation in CD4+ T lymphocytes and macrophages. Biology (Basel). 1, 94–115. 10.3390/biology1010094 24832049 PMC4011037

[B122] Rao V. R. Ruiz A. P. Prasad V. R. (2014). Viral and cellular factors underlying neuropathogenesis in HIV associated neurocognitive disorders (HAND). AIDS Res. Ther. 11:13. 10.1186/1742-6405-11-13 24894206 PMC4043700

[B123] Rappaport J. Joseph J. Croul S. Alexander G. Del Valle L. Amini S.. (1999). Molecular pathway involved in HIV-1-induced CNS pathology: role of viral regulatory protein, Tat. J. Leukoc. Biol. 65, 458–465. 10204574 10.1002/jlb.65.4.458

[B124] Robertson K. R. Smurzynski M. Parsons T. D. Wu K. Bosch R. J. Wu J.. (2007). The prevalence and incidence of neurocognitive impairment in the HAART era. AIDS 21, 1915–1921. 10.1097/QAD.0b013e32828e4e27 17721099

[B125] Robertson K. R. Su Z. Margolis D. M. Krambrink A. Havlir D. V. Evans S.. (2010). Neurocognitive effects of treatment interruption in stable HIV-positive patients in an observational cohort. Neurology 74, 1260–1266. 10.1212/WNL.0b013e3181d9ed09 20237308 PMC2860482

[B126] Rosinska M. Sieroslawski J. Wiessing L. (2015). High regional variability of HIV, HCV and injecting risks among people who inject drugs in Poland: comparing a cross-sectional bio-behavioural study with case-based surveillance. BMC Infect. Dis. 15:83. 10.1186/s12879-015-0828-9 25879904 PMC4340100

[B127] Sáez-Cirión A. Bacchus C. Hocqueloux L. Avettand-Fenoel V. Girault I. Lecuroux C.. (2013). Post-treatment HIV-1 controllers with a long-term virological remission after the interruption of early initiated antiretroviral therapy ANRS VISCONTI Study. PLoS Pathog. 9:e1003211. 10.1371/journal.ppat.1003211 23516360 PMC3597518

[B128] Saiyed Z. M. Gandhi N. Agudelo M. Napuri J. Samikkannu T. Reddy P. V.. (2011). HIV-1 Tat upregulates expression of histone deacetylase-2 (HDAC2) in human neurons: implication for HIV-associated neurocognitive disorder (HAND). Neurochem. Int. 58, 656–664. 10.1016/j.neuint.2011.02.004 21315782 PMC3085929

[B129] Scott J. C. Woods S. P. Carey C. L. Weber E. Bondi M. W. Grant I.. (2011). Neurocognitive consequences of HIV infection in older adults: an evaluation of the “cortical” hypothesis. AIDS Behav. 15, 1187–1196. 10.1007/s10461-010-9815-8 20865313 PMC3110599

[B130] Self R. L. Mulholland P. J. Harris B. R. Nath A. Prendergast M. A. (2004). Cytotoxic effects of exposure to the human immunodeficiency virus type 1 protein Tat in the hippocampus are enhanced by prior ethanol treatment. Alcohol. Clin. Exp. Res. 28, 1916–1924. 10.1097/01.ALC.0000148108.93782.05 15608609

[B131] Self R. L. Smith K. J. Butler T. R. Pauly J. R. Prendergast M. A. (2009). Intra-cornu ammonis 1 administration of the human immunodeficiency virus-1 protein trans-activator of transcription exacerbates the ethanol withdrawal syndrome in rodents and activates N-methyl-D-aspartate glutamate receptors to produce persisting spatial learning deficits. Neuroscience 163, 868–876. 10.1016/j.neuroscience.2009.07.025 19619615 PMC2773563

[B132] Sharma A. Hu X. T. Napier T. C. Al-Harthi L. (2011). Methamphetamine and HIV-1 Tat down regulate beta-catenin signaling: implications for methampetamine abuse and HIV-1 co-morbidity. J. Neuroimm. Pharmacol. 6, 597–607. 10.1007/s11481-011-9295-2 21744004 PMC3714216

[B133] Sharma H. S. Ali S. F. (2006). Alterations in blood-brain barrier function by morphine and methamphetamine. Ann. N.Y. Acad. Sci. 1074, 198–224. 10.1196/annals.1369.020 17105918

[B134] Siddappa N. B. Venkatramanan M. Venkatesh P. Janki M. V. Jayasuryan N. Desai A.. (2006). Transactivation and signaling functions of Tat are not correlated: biological and immunological characterization of HIV-1 subtype-C Tat protein. Retrovirology 3:53. 10.1186/1742-4690-3-53 16916472 PMC1564039

[B135] Silverstein P. S. Shah A. Gupte R. Liu X. Piepho R. W. Kumar S.. (2011). Methamphetamine toxicity and its implications during HIV-1 infection. J. Neurovirol. 17, 401–415. 10.1007/s13365-011-0043-4 21786077 PMC4118146

[B136] Simioni S. Cavassini M. Annoni J. M. Rimbault Abraham A. Bourquin I. Schiffer V.. (2010). Cognitive dysfunction in HIV patients despite long-standing suppression of viremia. AIDS 24, 1243–1250. 10.1097/QAD.0b013e3283354a7b 19996937

[B137] Smith D. B. Simmonds P. Bell J. E. (2014). Brain viral burden, neuroinflammation and neurodegeneration in HAART-treated HIV positive injecting drug users. J. Neurovirol. 20, 28–38. 10.1007/s13365-013-0225-3 24420447

[B138] Smurzynski M. Wu K. Letendre S. Robertson K. Bosch R. J. Clifford D. B.. (2011). Effects of central nervous system antiretroviral penetration on cognitive functioning in the ALLRT cohort. AIDS 25, 357–365. 10.1097/QAD.0b013e32834171f8 21124201 PMC3022370

[B139] Spira S. Wainberg M. A. Loemba H. Turner D. Brenner B. G. (2003). Impact of clade diversity on HIV-1 virulence, antiretroviral drug sensitivity and drug resistance. J. Antimicrob. Chemother. 51, 229–240. 10.1093/jac/dkg079 12562686

[B140] Stolbach A. Paziana K. Heverling H. Pham P. (2015). A review of the toxicity of HIV medications II: interactions with drugs and complementary and alternative medicine products. J. Med. Toxicol. 11, 326–341. 10.1007/s13181-015-0465-0 26036354 PMC4547966

[B141] Strazza M. Pirrone V. Wigdahl B. Nonnemacher M. R. (2011). Breaking down the barrier: the effects of HIV-1 on the blood-brain barrier. Brain Res. 1399, 96–115. 10.1016/j.brainres.2011.05.015 21641584 PMC3139430

[B142] Suzuki M. El-Hage N. Zou S. Hahn Y. K. Sorrell M. E. Sturgill J. L.. (2011). Fractalkine/CX3CL1 protects striatal neurons from synergistic morphine and HIV-1 Tat-induced dendritic losses and death. Mol. Neurodegener. 6:78. 10.1186/1750-1326-6-78 22093090 PMC3287119

[B143] Theodore S. Cass W. A. Maragos W. F. (2006b). Involvement of cytokines in human immunodeficiency virus-1 protein Tat and methamphetamine interactions in the striatum. Exp. Neurol. 199, 490–498. 10.1016/j.expneurol.2006.01.009 16510141

[B144] Theodore S. Cass W. A. Nath A. Maragos W. F. (2007). Progress in understanding basal ganglia dysfunction as a common target for methamphetamine abuse and HIV-1 neurodegeneration. Curr. HIV Res. 5, 301–313. 10.2174/157016207780636515 17504172

[B145] Theodore S. Cass W. A. Nath A. Steiner J. Young K. Maragos W. F. (2006a). Inhibition of tumor necrosis factor-alpha signaling prevents human immunodeficiency virus-1 protein Tat and methamphetamine interaction. Neurobiol. Dis. 23, 663–668. 10.1016/j.nbd.2006.05.005 16828290

[B146] Ti L. Milloy M. J. Shannon K. Simo A. Hogg R. S. Guillemi S.. (2014). Suboptimal plasma HIV-1 RNA suppression and adherence among sex workers who use illicit drugs in a Canadian setting: an observational cohort study. Sex. Transm. Infect. 90, 418–422. 10.1136/sextrans-2013-051408 24523347 PMC4102614

[B147] Toborek M. Lee Y. W. Flora G. Pu H. András I. E. Wylegala E.. (2005). Mechanisms of the blood-brain barrier disruption in HIV-1 infection. Cell. Mol. Neurobiol. 25, 181–199. 10.1007/s10571-004-1383-x 15962513 PMC11529531

[B148] Tong N. Perry S. W. Zhang Q. James H. J. Guo H. Brooks A.. (2000). Neuronal fractalkine expression in HIV-1 encephalitis: roles for macrophage recruitment and neuroprotection in the central nervous system. J. Immunol. 164, 1333–1339. 10.4049/jimmunol.164.3.1333 10640747

[B149] Tozzi V. Balestra P. Bellagamba R. Corpolongo A. Salvatori M. F. Visco-Comandini U.. (2007). Persistence of neuropsychologic deficits despite long-term highly active antiretroviral therapy in patients with HIV-related neurocognitive impairment: prevalence and risk factors. J. Acquir. Immune Defic. Syndr. 45, 174–182. 10.1097/QAI.0b013e318042e1ee 17356465

[B150] Tozzi V. Balestra P. Galgani S. Narciso P. Ferri F. Sebastiani G.. (1999). Positive and sustained effects of highly active antiretroviral therapy on HIV-1-associated neurocognitive impairment. AIDS 13, 1889–1897. 10.1097/00002030-199910010-00011 10513647

[B151] Turchan-Cholewo J. Dimayuga F. O. Ding Q. Keller J. N. Hauser K. F. Knapp P. E.. (2008). Cell-specific actions of HIV-Tat and morphine on opioid receptor expression in glia. J. Neurosci. Res. 86, 2100–2110. 10.1002/jnr.21653 18338799 PMC2760290

[B152] Wadia R. S. Pujari S. N. Kothari S. Udhar M. Kulkarni S. Bhagat S.. (2001). Neurological manifestations of HIV disease. J. Assoc. Physicians India 49, 343–348. 11291974

[B153] Wang T. Rumbaugh J. A. Nath A. (2006). Viruses and the brain: from inflammation to dementia. Clin. Sci. 110, 393–407. 10.1042/CS20050278 16526945

[B154] Weiss N. Miller F. Cazaubon S. Couraud P. O. (2009). The blood-brain barrier in brain homeostasis and neurological diseases. Biochim. Biophys. Acta 1788, 842–857. 10.1016/j.bbamem.2008.10.022 19061857

[B155] Wires E. S. Alvarez D. Dobrowolski C. Wang Y. Morales M. Karn J.. (2012). Methamphetamine activates nuclear factor kappa-light-chain-enhancer of activated B cells (NF-kappaB) and induces human immunodeficiency virus (HIV) transcription in human microglial cells. J. Neurovirol. 18, 400–410. 10.1007/s13365-012-0103-4 22618514 PMC3469781

[B156] Woollard S. M. Bhargavan B. Yu F. Kanmogne G. D. (2014). Differential effects of Tat proteins derived from HIV-1 subtypes B and recombinant CRF02_AG on human brain microvascular endothelial cells: implications for blood-brain barrier dysfunction. J. Cereb. Blood Flow Metabol. 34, 1047–1059. 10.1038/jcbfm.2014.54 24667918 PMC4050250

[B157] Xu R. Feng X. Xie X. Zhang J. Wu D. Xu L. (2012). HIV-1 Tat protein increases the permeability of brain endothelial cells by both inhibiting occludin expression and cleaving occludin via matrix metalloproteinase-9. Brain Res. 1436, 13–19. 10.1016/j.brainres.2011.11.052 22197032

[B158] Yukl S. Pillai S. Li P. Chang K. Pasutti W. Ahlgren C.. (2009). Latently-infected CD4+ T cells are enriched for HIV-1 Tat variants with impaired transactivation activity. Virology 387, 98–108. 10.1016/j.virol.2009.01.013 19268337 PMC4474533

[B159] Zhong Y. Zhang B. Eum S. Y. Toborek M. (2012). HIV-1 Tat triggers nuclear localization of ZO-1 via Rho signaling and cAMP response element-binding protein activation. J. Neurosci. 32, 143–150. 10.1523/JNEUROSCI.4266-11.2012 22219277 PMC3566645

